# Fear of COVID-19, healthy eating behaviors, and health-related behavior changes as associated with anxiety and depression among medical students: An online survey

**DOI:** 10.3389/fnut.2022.938769

**Published:** 2022-09-23

**Authors:** Minh H. Nguyen, Tinh X. Do, Tham T. Nguyen, Minh D. Pham, Thu T. M. Pham, Khue M. Pham, Giang B. Kim, Binh N. Do, Hiep T. Nguyen, Ngoc-Minh Nguyen, Hoa T. B. Dam, Yen H. Nguyen, Kien T. Nguyen, Thao T. P. Nguyen, Trung T. Nguyen, Tuyen Van Duong

**Affiliations:** ^1^International Ph.D. Program in Medicine, College of Medicine, Taipei Medical University, Taipei, Taiwan; ^2^Department of Psychiatry, Military Hospital 103, Hanoi, Vietnam; ^3^Faculty of Public Health, Hai Phong University of Medicine and Pharmacy, Hai Phong, Vietnam; ^4^Department of Clinical Nutrition, Military Hospital 103, Hanoi, Vietnam; ^5^Department of Nutrition, Vietnam Military Medical University, Hanoi, Vietnam; ^6^School of Public Health, College of Public Health, Taipei Medical University, Taipei, Taiwan; ^7^President Office, Hai Phong University of Medicine and Pharmacy, Hai Phong, Vietnam; ^8^Institute of Preventive Medicine and Public Health, Hanoi Medical University, Hanoi, Vietnam; ^9^Center for Assessment and Quality Assurance, Hanoi Medical University, Hanoi, Vietnam; ^10^Department of Infectious Diseases, Vietnam Military Medical University, Hanoi, Vietnam; ^11^Division of Military Science, Military Hospital 103, Hanoi, Vietnam; ^12^Faculty of Public Health, Pham Ngoc Thach University of Medicine, Ho Chi Minh, Vietnam; ^13^Pham Ngoc Thach Clinic, Pham Ngoc Thach University of Medicine, Ho Chi Minh, Vietnam; ^14^President Office, Pham Ngoc Thach University of Medicine, Ho Chi Minh, Vietnam; ^15^Department of Psychiatry, Thai Nguyen University of Medicine and Pharmacy, Thai Nguyen, Vietnam; ^16^Department of Pharmacology and Clinical Pharmacy, Can Tho University of Medicine and Pharmacy, Can Tho, Vietnam; ^17^Department of Pharmacy, Can Tho University of Medicine and Pharmacy Hospital, Can Tho, Vietnam; ^18^President Office, Can Tho University of Medicine and Pharmacy, Can Tho, Vietnam; ^19^Department of Physiology, Faculty of Medicine, Can Tho University of Medicine and Pharmacy, Can Tho, Vietnam; ^20^Institute for Community Health Research, University of Medicine and Pharmacy, Hue University, Hue, Vietnam; ^21^School of Medicine and Pharmacy, Vietnam National University, Hanoi, Vietnam; ^22^School of Nutrition and Health Sciences, Taipei Medical University, Taipei, Taiwan

**Keywords:** fear, COVID-19, smoking, physical activity, healthy eating behavior, anxiety, depression, medical students

## Abstract

**Background:**

Medical students' health and wellbeing are highly concerned during the COVID-19 pandemic. This study examined the impacts of fear of COVID-19 (FCoV-19S), healthy eating behavior, and health-related behavior changes on anxiety and depression.

**Methods:**

We conducted an online survey at 8 medical universities in Vietnam from 7th April to 31st May 2020. Data of 5,765 medical students were collected regarding demographic characteristics, FCoV-19S, health-related behaviors, healthy eating score (HES), anxiety, and depression. Logistic regression analyses were used to explore associations.

**Results:**

A lower likelihood of anxiety and depression were found in students with a higher HES score (OR = 0.98; 95%CI = 0.96, 0.99; *p* = 0.042; OR = 0.98; 95%CI = 0.96, 0.99; *p* = 0.021), and in those unchanged or more physical activities during the pandemic (OR = 0.54; 95%CI = 0.44, 0.66; *p* < 0.001; OR = 0.44; 95%CI = 0.37, 0.52; *p* < 0.001) as compared to those with none/less physical activity, respectively. A higher likelihood of anxiety and depression were reported in students with a higher FCoV-19S score (OR = 1.09; 95%CI = 1.07, 1.12; *p* < 0.001; OR = 1.06; 95%CI = 1.04, 1.08; *p* < 0.001), and those smoked unchanged/more during the pandemic (OR = 6.67; 95%CI = 4.71, 9.43; *p* < 0.001; OR = 6.77; 95%CI = 4.89, 9.38; *p* < 0.001) as compared to those stopped/less smoke, respectively. In addition, male students had a lower likelihood of anxiety (OR = 0.79; 95%CI = 0.65, 0.98; *p* = 0.029) compared to female ones.

**Conclusions:**

During the pandemic, FCoV-19S and cigarette smoking had adverse impacts on medical students' psychological health. Conversely, staying physically active and having healthy eating behaviors could potentially prevent medical students from anxiety and depressive symptoms.

## Introduction

The COVID-19 pandemic has been causing the ever-increasing number of confirmed cases and deaths worldwide (1), placing a huge burden on the healthcare system (2–4). Amidst the pandemic, healthcare workers (HCWs) have directly involved in providing care and treatment for COVID-19 patients. The HCWs have faced stressful challenges, including lack of experience, overwhelming workload, shortages of personal protective equipment, and fear of contagion for their loved ones (5–7). Besides, long working hours and under high-pressure situations make HCWs physically and mentally exhausted which may also increase the risk of infection. In addition to frontline HCWs, most medical staff, regardless of their profession, have experienced remarkable changes in their working conditions and time, and a lack of interaction with colleagues and patients (8). These factors may significantly contribute to the development of psychological burdens among healthcare staff. Recent scientific literature also showed that HCWs were at increased risk of developing psychological illnesses during the COVID-19 crisis (7, 9, 10). Medical students are the future health workforce for the medical system, they had significant roles in containing the COVID-19 pandemic (11–15), their mental health requires more attention and additional support.

Unlike students studying in other disciplines, medical students have been reported to have a greater risk of developing psychological disorders during the pandemic (16–18). Before the pandemic, factors influencing their mental health were documented, including heavy academic programs, rigorous exams, work-life imbalance, the difficulty in adapting to clinical environments and exposing to critically ill and dying patients (19, 20). During the pandemic, Vietnam has adopted various preventive measures to control the spread of COVID-19, including a nationwide lockdown from April 1–20, 2020 (21, 22). Since educational institutions had to be closed for a long time (23), education programs transitioned from the classroom learning to the online learning (24). Meanwhile, medical students have to perform practical training, online studying may affect their academic progress. Besides, prevention measures (e.g., lockdown, and home quarantine) could cause feelings of isolation, leading to harmful lifestyles (25–27). These factors may have negative impacts on their psychological health. Therefore, it is essential to understand the impact of COVID-19 on the mental health of medical students, thereby promoting appropriate strategies to help them reduce the risk of developing mental disorders during the pandemic.

The uncertainty of the COVID-19 pandemic can cause increased fear in the community. Previous studies indicated that there were high rates of anxiety, depression, and psychological distress in the general population during the COVID-19 pandemic (28–30). In addition, COVID-19-related fear was found to be positively associated with mental disorders (31, 32). Particularly, medical students have a better knowledge of the disease and its severity, making them more fearful and worried during the pandemic (33). Therefore, anxiety and depression in relation to the fear of COVID-19 should be investigated in medical students to assess their mental health.

Engaging in positive lifestyles (e.g., healthy diet, staying physically active, avoiding alcohol and smoking) were documented to have benefits for mental health (34, 35). However, when facing the unfamiliar situations that the COVID-19 pandemic and preventive measures (e.g., lockdown), people had an increased tendency to consume alcohol and smoke cigarettes as a coping method (36–38). In addition, a recent systematic review of 87 articles indicated that there were increased food and alcohol consumption, and increased sedentary hours during the COVID-19 pandemic (39, 40). Another research also showed that a higher intake of unhealthy foods was documented during the lockdown period (40). The changes of lifestyle may further affect people's psychological health. Therefore, health-related behaviors should be investigated as independent variables for medical student's mental health.

Previous literature indicated that the prevalence of anxiety and depression varied across demographic characteristics, such as age, gender, income, education (41, 42). In the context of the pandemic, several health-related factors were reported to be important predictors of mental disorders, such as underlying health conditions, symptoms like COVID-19, or BMI (41–43). In addition, recent studies also showed that higher digital healthy diet literacy (DDL) and health literacy (HL) were associated with better mental health in different populations (e.g., healthcare workers, and outpatients) (43–45). Therefore, DDL and HL may have potential impacts on anxiety and depression among medical students during the pandemic.

This study was conducted to examine the associated factors of anxiety and depression among undergraduate medical students, in which impacts of fear of COVID-19, health-related behavior changes and healthy eating behaviors were emphasized.

## Methods

### Study design and sample

We conducted a cross-sectional study among medical students at 8 medical universities across Vietnam, including 5 universities in the Northern area, 1 university in the Central area, and 2 universities in the Southern area. An online questionnaire survey was carried out to collect data from 7^th^ April to 31^st^ May 2020. A sample of 5,765 students (out of 28,737 possible students) completed the online survey (46).

This study received ethical approval from the Institutional Ethical Review Committee of Hanoi University of Public Health, Vietnam (IRB No. 133/2020/YTCC-HD3).

We used a convenience sampling method to recruit medical students. Researchers (university lecturers) informed and invited their students to participate in the survey. Next, the online survey link was sent to student leaders, who were responsible for sharing the link with other students in their class *via* email, Facebook messenger, or Zalo. Study purposes were informed to students before they signed the electronic consent forms. Students then completed all the survey questions. There was no missing data as marking required fields for all the questions. Students' data is kept confidential and used for research purposes only.

### Measurements

#### Outcome variables

Anxiety and depression were assessed using the 7-item Generalized Anxiety Disorder (GAD-7) questionnaire and the 9-item Patient Health Questionnaire (PHQ-9), respectively. The original translated versions of GAD-7 and PHQ-9 were used in this study and in the Vietnamese context (47–49). In the current study, the Cronbach's alpha values for the GAD-7 and PHQ-9 questionnaires were 0.94 and 0.91, respectively. These questionnaires investigate participants about the extent to which different symptoms of anxiety and depression bothered them in the last 2 weeks with four possible responses from “0 = not at all” to “3 = almost every day”. A sum GAD-7 score (a range of 0–21) of ≥ 8 was classified as anxiety (50). Similarly, a sum PHQ-9 score (a range of 0–27) of ≥ 10 was classified as depression (51).

#### Fear of COVID-19

We used the 7-item fear of COVID-19 scale (FCoV-19S) to evaluate fear. This instrument was validated and used in Vietnamese medical students in a previous study (52). Students were asked to rate their agreements with varying degrees of the COVID-19 related fear. The possible answers range from “1 = strongly disagree” to “5 = strongly agree”. The sum scores varied between 7 and 35, in which students with higher scores have greater degrees of FCoV-19S.

#### Healthy eating behavior

We used the healthy eating score (HES-5) questionnaire to evaluate healthy eating behavior (53, 54). This instrument consists of 5 food items, which investigate how often students consumed these foods in the last 30 days, including vegetables, fruits, fish, whole grains, and dairy products. The frequency of food consumption ranges from 0 (rarely or never) to 5 (three or more times every day). The sum score of HES is between 0 and 25, in which students with higher scores have healthier eating habits. This tool was validated and used for assessing a healthy diet in different Vietnamese populations (44, 47). The Cronbach's alpha for HES-5 in the current study was 0.73.

#### Health-related behavior changes

Students reported their health behaviors amidst the pandemic compared to the pre-pandemic, including cigarette smoking, alcohol drinking, physical activity with five choices (never, quitted/stopped, less, unchanged, and more); and eating habits with 3 choices (less healthy, unchanged, and healthier). It is recommended that people should maintain or make better positive behaviors (e.g., physical activity, healthy diet) to stay healthy during the pandemic. Inversely, harmful behaviors (e.g., smoking, drinking) should be abandoned or reduced gradually (55). Therefore, we classified health-related behavior changes into two categories as follows: cigarette smoking (“none or smoke less” vs. “unchanged or smoke more”), alcohol drinking (“none or drink less” vs. “unchanged or drink more”), physical activity (“none or less active” vs. “unchanged or more active”) and eating habits (“unchanged or less healthy” vs. “healthier”).

### Digital healthy diet literacy and health literacy

We used the 4-item digital healthy diet literacy questionnaire (DDL-4) and 12-item short-form health literacy questionnaire (HLS-SF12) to evaluate student's digital healthy diet literacy (DDL) and health literacy (HL). The DDL-4 was developed, validated, and used in previous studies during the pandemic (44, 46, 56), while the HLS-SF12 was commonly utilized for research in Asian nations (57) and Vietnam (58–62). The Cronbach's alpha for the DDL-4 and HLS-SF12 in our study were 0.87 and 0.89, respectively. Students rated their performance difficulty for each questionnaire item on four-level responses ranging from “1 = very difficult” to “4 = very easy”. We standardized the DDL and HL scores into unified metrics ranging from 0 to 50, in which students with higher DDL scores or higher HL scores have better DDL or HL. The standardized formula was represented in previous research (46, 57).

### Socio-demographic and clinical characteristics

Data related to student's characteristics were also collected, including age, sex (female vs. male), ability to pay for medical care (easy vs. difficult), and academic year (1–2 vs. 3–6). Bodyweight (kg) and height (cm) were self-reported by students, which were used to calculate body mass index (BMI, kg/m^2^). We used the Charlson Comorbidity Index items (63) to evaluate underlying health conditions (none vs. one or more). Students also reported Suspected COVID-19 symptoms (S-COVID-19-S) that they had at the time of the survey. These symptoms include fever, cough, difficult breathing, myalgia, fatigue, sputum production, confusion, headache, sore throat, rhinorrhea, chest pain, hemoptysis, diarrhea, and nausea (64). Students had S-COVID-19-S if they had at least one of S-COVID-19-S.

### Data analysis

First, descriptive analyses were used to summarize the features of independent variables (IVs), including frequency, percentage, mean, and standard deviation. Second, we used the Chi-squared test to compare the distribution of anxiety and depression according to different groups of IVs. Third, simple and multiple logistic regression models were conducted to explore the associated predictors of anxiety and depression in medical students. We chose variables related to outcomes at *p* < 0.1 in bivariate models to perform multivariate models. The Spearman correlation test was utilized to test relationships between IVs to deal with multicollinearity. We found that age highly correlates with the academic year (*rho* = 0.82); cigarette smoking moderately correlates with alcohol drinking (*rho* = 0.49); health literacy moderately correlates with the DDL (*rho* = 0.63) (Supplementary Table S1). Thus, age, gender, cigarette smoking, health literacy, and other independent factors were included in the multiple logistic regression models. We set *p-value* < 0.05 as a significant level. All analyses were performed using the IBM SPSS Version 26.0 (IBM Corp, Armonk, NY, United States).

## Results

### Student's characteristics

The average age of the sample was 21.7 ± 1.9. Out of all participants, 47.3% were female, 46.3% responded to difficult payment for medical care, and 35.3% were first-year or second-year students. The mean score of FCoV-19S was 16.6 ± 5.2. The prevalence of anxiety and depression was 8.1 and 12.2%, respectively. The proportion of anxiety varied by different groups of gender, ability to pay for medical care, S-COVID-19-S, underlying health conditions, cigarette smoking, alcohol drinking, physical activity, and eating habits. The proportion of depression varied by different groups of ability to pay for medical care, S-COVID-19-S, underlying health conditions, cigarette smoking, alcohol drinking, physical activity, and eating habits (Table 1).

**Table 1:** Characteristics of medical students by anxiety and depression (*n* = 5765).

**Variables**	**Total** ** (*n =* 5765)**	**Anxiety disorders**			**Depressive symptoms**		
		**GAD < 8** **(*n =* 5298)**	**GAD ≥ 8** ** (*n =* 467)**		**PHQ < 10** ** (*n =* 5061)**	**PHQ ≥10** ** (*n =* 704)**	
	***n* (%)**	***n* (%)**	***n* (%)**	p*	***n* (%)**	***n* (%)**	p*
Age, year (mean ± SD)	21.7 ± 1.9	**-**	**-**	**-**	**-**	**-**	**-**
Gender				0.001			0.089
Female	2,726 (47.3)	2,470 (90.6)	256 (9.4)		2,372 (87.0)	354 (13.0)	
Male	3,039 (52.7)	2,828 (93.1)	211 (6.9)		2,689 (88.5)	350 (11.5)	
Ability to pay for medical care				< 0.001			< 0.001
Very or fairly easy	3,096 (53.7)	2,397 (89.8)	272 (10.2)		2,257 (84.6)	412 (15.4)	
Very or fairly difficult	2,669 (46.3)	2,901 (93.7)	195 (6.3)		2,804 (90.6)	292 (9.4)	
Academic year				0.834			0.825
1–2	2,036 (35.3)	1,869 (91.8)	167 (8.2)		1,790 (87.9)	246 (12.1)	
3–6	3,729 (64.7)	3,429 (92.0)	300 (8.0)		3,271 (87.7)	458 (12.3)	
Suspected COVID-19 symptoms				< 0.001			< 0.001
No	4,695 (81.4)	4,376 (93.2)	319 (6.8)		4,205 (89.6)	490 (10.4)	
Yes	1,070 (18.6)	922 (86.2)	148 (13.8)		856 (80.0)	214 (20.0)	
Underlying health conditions				< 0.001			< 0.001
None	5,517 (95.7)	5,096 (92.4)	421 (7.6)		4,878 (88.4)	639 (11.6)	
One or more	248 (4.3)	202 (81.5)	46 (18.5)		183 (73.8)	65 (26.2)	
BMI, kg/m^2^				0.228			0.066
Normal weight (BMI < 25.0)	5,313 (92.2)	4,889 (92.0)	424 (8.0)		4,676 (88.0)	637 (12.0)	
Overweight/obese (BMI ≥25.0)	448 (7.8)	405 (90.4)	43 (9.6)		381 (85.0)	67 (15.0)	
Cigarette smoking				< 0.001			< 0.001
None or smoke less	5,577 (96.7)	5,180 (92.9)	397 (7.1)		4,957 (88.9)	620 (11.1)	
Unchanged or smoke more	188 (3.3)	118 (62.8)	70 (37.2)		104 (55.3)	84 (44.7)	
Alcohol drinking				< 0.001			< 0.001
None or drink less	5,346 (92.7)	4,967 (92.9)	379 (7.1)		4,749 (88.8)	597 (11.2)	
Unchanged or drink more	419 (7.3)	331 (79.0)	88 (21.0)		312 (74.5)	107 (25.5)	
Physical activity				< 0.001			< 0.001
None or less active	1,810 (31.4)	1,607 (88.8)	203 (11.2)		1,478 (81.7)	332 (18.3)	
Unchanged or more active	3,955 (68.6)	3691 (93.3)	264 (6.7)		3583 (90.6)	372 (9.4)	
Eating habits				0.003			0.002
Unchanged or less healthy	3,429 (59.5)	3,121 (91.0)	308 (9.0)		2,973 (86.7)	456 (13.3)	
Healthier	2,336 (40.5)	2,177 (93.2)	159 (6.8)		2,088 (89.4)	248 (10.6)	
Healthy eating score, mean ± SD	14.5 ± 4.7	**-**	**-**	**-**	**-**	**-**	**-**
Fear of COVID-19 score, mean ± SD	16.6 ± 5.2	**-**	**-**	**-**	**-**	**-**	**-**
Health literacy index, mean ± SD	34.6 ± 7.0	**-**	**-**	**-**	**-**	**-**	**-**
Digital healthy diet literacy, mean ± SD	34.0 ± 8.7	**-**	**-**	**-**	**-**	**-**	**-**

PHQ, patient health questionnaire; GAD, Generalized Anxiety Disorder; SD, standard deviation; BMI, Body Mass Index.

^*^Results of the Chi-square test.

### Associated factors of anxiety among medical students

The multiple logistic regression models show that medical students had a lower likelihood of anxiety were male (odds ratio, OR: 0.79; 95% confidence interval, 95% CI: 0.65, 0.98; *p* = 0.029), those with higher healthy eating scores (OR: 0.98; 95% CI: 0.96, 0.99; *p* = 0.042) and those with unchanged or more physical activity (OR: 0.54; 95%CI: 0.44, 0.66; *p* < 0.001) as compared to those with none or less physical activity during the pandemic. Whereas, medical students had a higher likelihood of anxiety were those who found it difficult to pay for medical care (OR: 1.55; 95% CI: 1.27, 1.90; *p* < 0.001), those with symptoms like COVID-19 (OR: 2.11; 95% CI: 1.69, 2.63; *p* < 0.001), those with one one more underlying health conditions (OR: 2.45; 95% CI: 1.71, 3.53; *p* < 0.001), those with unchanged or more smoking during the pandemic (OR: 6.67; 95% CI: 4.71, 9.43; *p* < 0.001), and those with higher fear of COVID-19 scores (OR: 1.09; 95% CI: 1.07, 1.12; *p* < 0.001), as compared to their counterparts (Table 2).

**Table 2:** Factors associated with anxiety disorders among medical students (*n* = 5765).

**Variables**	**Anxiety disorders**			
	**Simple regression**		**Multiple regression**	
	**OR (95% CI)**	***p***	**OR (95% CI)**	***p***
Age	0.98 (0.93, 1.03)	0.464	0.99 (0.94, 1.05)	0.758
**Gender**				
Female	Ref.		Ref.	
Male	0.72 (0.59, 0.87)	0.001	0.79 (0.65, 0.98)	0.029
**Ability to pay for medical care**				
Very or fairly easy	Ref.		Ref.	
Very or fairly difficult	1.69 (1.39, 2.04)	< 0.001	1.55 (1.27, 1.90)	< 0.001
**Academic year**				
1–2	Ref.		-	-
3–6	0.98 (0.80, 1.19)	0.834	-	-
**Suspected COVID-19 symptoms**				
No	Ref.		Ref.	
Yes	2.20 (1.79, 2.71)	< 0.001	2.11 (1.69, 2.63)	< 0.001
**Underlying health conditions**				
None	Ref.		Ref.	
One or more	2.76 (1.97, 3.85)	< 0.001	2.45 (1.71, 3.53)	< 0.001
**BMI, kg/m** ^**2**^				
Normal weight (BMI < 25.0)	Ref.		-	-
Overweight/obese (BMI ≥25.0)	1.22 (0.88, 1.70)	0.229	-	-
**Cigarette smoking**				
None or smoke less	Ref.		Ref.	
Unchanged or smoke more	7.74 (5.66, 10.58)	< 0.001	6.67 (4.71, 9.43)	< 0.001
**Alcohol drinking**				
None or drink less	Ref.		-	-
Unchanged or drink more	3.48 (2.69, 4.51)	< 0.001	-	-
**Physical activity**				
None or less active	Ref.		Ref.	
Unchanged or more active	0.57 (0.47, 0.69)	< 0.001	0.54 (0.44, 0.66)	< 0.001
**Eating habits**				
Unchanged or less healthy	Ref.		Ref.	
Healthier	0.74 (0.61, 0.90)	0.003	0.87 (0.70, 1.08)	0.200
Healthy Eating Score, 1 score increment	0.97 (0.95, 0.99)	0.004	0.98 (0.96, 0.99)	0.042
Fear of COVID-19 score, 1 score increment	1.11 (1.09, 1.14)	< 0.001	1.09 (1.07, 1.12)	< 0.001
Health literacy index, 1 score increment	0.98 (0.97, 1.00)	0.092	0.99 (0.98, 1.01)	0.681
Digital healthy diet literacy, 1 score increment	0.99 (0.98, 1.00)	0.165	-	-

GAD, Generalized Anxiety Disorder; OR, odds ratio; CI, confidence interval; BMI, Body Mass Index.

### Associated factors of depression among medical students

The multiple logistic regression models indicate that medical students had lower odds of depression were those with higher healthy eating scores (OR: 0.98; 95% CI: 0.96, 0.99; *p* = 0.021), those with unchanged or more physical activity (OR: 0.44; 95% CI: 0.37, 0.52; *p* < 0.001) as compared to those with none or less physical activity during the pandemic. Whereas, medical students had higher odds of depression were those who found it difficult to pay for medical care (OR: 1.62; 95% CI: 1.37, 1.92; *p* < 0.001), those with COVID-19-like symptoms (OR: 2.00; 95% CI: 1.66, 2.42; *p* < 0.001), those with one one more underlying health conditions (OR: 2.50; 95% CI: 1.82, 3.43; *p* < 0.001), those with unchanged or more smoking during the pandemic (OR: 6.77; 95% CI: 4.89, 9.38; *p* < 0.001), those with higher fear of COVID-19 scores (OR: 1.06; 95% CI: 1.04, 1.08; *p* < 0.001), as compared to their counterparts (Table 3).

**Table 3:** Factors associated with depression among medical students (*n* = 5765).

**Variables**	**Depressive symptoms**			
	**Simple regression**		**Multiple regression**	
	**OR (95% CI)**	***p***	**OR (95% CI)**	***p***
Age	1.00 (0.96, 1.04)	0.967	1.01 (0.96, 1.05)	0.755
**Gender**				
Female	Ref.		Ref.	
Male	0.87 (0.74, 1.02)	0.089	0.96 (0.81, 1.15)	0.684
**Ability to pay for medical care**				
Very or fairly easy	Ref.		Ref.	
Very or fairly difficult	1.75 (1.49, 2.06)	< 0.001	1.62 (1.37, 1.92)	< 0.001
**Academic year**				
1–2	Ref.		-	-
3–6	1.02 (0.86, 1.20)	0.825	-	-
**Suspected COVID-19 symptoms**				
No	Ref.		Ref.	
Yes	2.14 (1.79, 2.56)	< 0.001	2.00 (1.66, 2.42)	< 0.001
**Underlying health conditions**				
None	Ref.		Ref.	
One or more	2.71 (2.02, 3.64)	< 0.001	2.50 (1.82, 3.43)	< 0.001
**BMI, kg/m** ^**2**^				
Normal weight (BMI < 25.0)	Ref.		Ref.	
Overweight/obese (BMI ≥25.0)	1.29 (0.98, 1.69)	0.066	1.17 (0.87, 1.57)	0.302
**Cigarette smoking**				
None or smoke less	Ref.		Ref.	
Unchanged or smoke more	6.46 (4.79, 8.71)	< 0.001	6.77 (4.89, 9.38)	< 0.001
**Alcohol drinking**				
None or drink less	Ref.		-	-
Unchanged or drink more	2.73 (2.15, 3.45)	< 0.001	-	-
**Physical activity**				
None or less active	Ref.		Ref.	
Unchanged or more active	0.46 (0.39, 0.54)	< 0.001	0.44 (0.37, 0.52)	< 0.001
**Eating habits**				
Unchanged or less healthy	Ref.		Ref.	
Healthier	0.77 (0.66, 0.91)	0.002	0.95 (0.79, 1.14)	0.563
Healthy Eating Score, 1 score increment	0.97 (0.95, 0.98)	< 0.001	0.98 (0.96, 0.99)	0.021
Fear of COVID-19 score, 1 score increment	1.07 (1.05, 1.09)	< 0.001	1.06 (1.04, 1.08)	< 0.001
Health literacy score, 1 score increment	0.98 (0.97, 0.99)	< 0.001	0.99 (0.98, 1.01)	0.105
Digital healthy diet literacy, 1 score increment	0.98 (0.97, 0.99)	0.002	-	-

PHQ, Patient Health Questionnaire; OR, odds ratio; CI, confidence interval; BMI, Body Mass Index.

## Discussion

In this study, our results indicate that medical students with higher fear of COVID-19 were more likely to have mental disorders. Our finding was consistent with recent literature conducted in China, Turkey, and the United Arab Emirates among university students (65–67). It is understandable that medical students have a better knowledge of the disease, making them more aware of the severity and danger of the virus. Especially, students in clinical training years are required to work in teaching hospitals and emergency units, which are high-risk environments. Therefore, students may feel more anxious and depressed because they are at higher risk of getting infected and may pass the virus on to loved ones. This explanation also partially elucidates the result of this study that students with symptoms resembling COVID-19 were prone to be anxious and depressed. Previous studies conducted on different populations, such as outpatients, HCWs also reported similar results (41, 43, 48). In addition, we found that underlying health conditions were positively associated with anxiety and depression, which was in line with the findings of recent studies (42, 48, 68). The explanation for this association is that medical students have a better understanding of the disease. Therefore, they know that underlying medical conditions may worsen health outcomes after COVID-19 infections (69), making students with comorbidities more anxious and depressed during the period of the pandemic. Thus, psychological supports that mitigate the fear and enhance mental resilience could be essential to prevent medical students from developing anxiety or depressive symptoms.

The result of this study shows that medical students who smoked unchanged or more during the pandemic were more likely to be anxious and depressed. Our result was in line with previous studies conducted during the pandemic (49, 70). It is reported that smoking is quite common among medical students, especially among male students (71, 72). Although, smoking could help to relieve stress and pressure when facing uncomfortable events, especially in hospital settings. However, the long-term effects of smoking on the development of later anxiety and depression have been demonstrated in various studies (73). In addition, the present study found that maintaining physical activity unchanged or more active during the pandemic could help to prevent medical students from mental disorders. Similar findings were found in other studies carried out in Australia, North America, Brazil, and China among different populations during the pandemic (70, 74–76). Furthermore, physical activity was highly recommended for depression treatment (77). Regular exercise could help to enhance immune function (78–80), which prevents the body from pathogens, improving physical and mental health during the pandemic. This study also highlights the role of healthy eating behaviors in protecting medical students against the development of anxiety and depressive symptoms. The beneficial effect of a healthy diet on psychological health was reported in recent literature (35, 47, 48). Recent research on college students carried out during a home-confinement period in Italy indicated that healthy dietary behaviors were negatively linked with poorer mental states (81). However, restrictions to outdoor activities and daily lives caused by the COVID-19 pandemic may lead to weight gain, and adversely influence eating habits, and sleeping patterns, thereby increasing eating disorder risk and symptoms (82, 83). In addition, due to precaution measures applied during the pandemic, people were more likely to engage in harmful lifestyles (e.g., increased screen time and sedentary behaviors, or increased alcohol consumption) (25, 39, 40). Therefore, our findings related to health-related behaviors provide timely evidence that helps to encourage medical school students to engage in positive lifestyle habits, reducing the likelihood of mental disorders during the pandemic.

Moreover, we found that male medical students had a lower likelihood of developing anxiety than their female colleagues. This result is similar to previous studies conducted in the United States, Bangladeshi among medical students (84, 85). Our study also indicated that medical students who responded to payment difficulty for medical care had higher odds of anxiety and depressive symptoms than their counterparts, which is comparable to the findings of other studies conducted on outpatients and HCWs during the pandemic (47, 49). The difficult affordability of healthcare may lead to delays in examinations and treatments, which negatively affect students' physical and mental health. It may also partially reflex the financial constraints (86). Particularly, the unemployment rate was higher during the pandemic, affecting the household income (87, 88), making medical students more worried and stressed about the cost of living, rent, and education. Therefore, medical universities should consider strategies to assess and support the psychological health of vulnerable demographic groups, including medical students who are female and who find it difficult to pay for healthcare.

Our study was strengthened by its large sample size collected from 8 medical universities across Vietnam. It is also the first study to evaluate the impact of COVID-19 on mental health among medical students in Vietnam. Therefore, this pilot study could provide timely evidence for future research and practices to protect mental health against the adverse impact of the COVID-19 pandemic. However, some limitations need to be acknowledged in this paper. First, the causality of the associations could not be inferred in the study with a cross-sectional design. Second, as students recruited in the survey were not randomly selected, the generalization of these results should be applied cautiously to medical school students. Final, several factors, which may affect outcomes, were not investigated in this study, such as the changes in teaching methods, financial problems, history of mental disorders, relationships with friends and family, and academic workload. Future studies should take these variables into consideration in assessments.

## Conclusions

Amidst the COVID-19 pandemic, medical students with higher fear scores were more likely to have anxiety and depression. Students who smoked had a higher likelihood of having anxiety and depression. Fortunately, staying physically active and having healthy eating behavior were found to be protective factors of mental health among medical students. Medical universities should develop strategic programs to encourage students to actively engage in physical activity, healthy diet, and avoid smoking, which could prevent medical students from psychological disorders during the pandemic.

## Data availability statement

The raw data supporting the conclusions of this article will be made available on reasonable request to the corresponding author.

## Ethics statement

The studies involving human participants were reviewed and approved by the Institutional Ethical Review Committee of Hanoi University of Public Health, Vietnam (IRB No. 133/2020/YTCC-HD3). The patients/participants provided their written informed consent to participate in this study.

## Author contributions

MHN, TXD, ThTN, MDP, TTMP, KMP, GBK, BND, HTN, N-MN, HTBD, YHN, KTN, TTPN, TrTN, and TVD: conceptualization, methodology, validation, investigation, data curation, and writing-review and editing draft. MHN, TD, and TD: formal analysis and writing-original draft. MHN, TTMP, and TTPN: project administration. TD: funding acquisition, supervision. All authors have read and approved the final manuscript.

## Funding

This research was funded by Taipei Medical University and Military Hospital 103 (108-6202-008-112; 108-3805-022-400).

## Conflict of interest

The authors declare that the research was conducted in the absence of any commercial or financial relationships that could be construed as a potential conflict of interest.

## Publisher's note

All claims expressed in this article are solely those of the authors and do not necessarily represent those of their affiliated organizations, or those of the publisher, the editors and the reviewers. Any product that may be evaluated in this article, or claim that may be made by its manufacturer, is not guaranteed or endorsed by the publisher.
