# Supplementary material for: Fear of COVID-19, healthy eating behaviors, and health-related behavior changes as associated with anxiety and depression among medical students: An online survey
Source: Front Nutr. 2022 Sep 23;9:938769. doi: 10.3389/fnut.2022.938769 (PMC9538633; doi:10.3389/fnut.2022.938769)
Supplement: Supplementary file 1 [file Table_1.DOCX]

**Fear of COVID-19, Healthy Eating Behavior, and Health-related Behavior Changes as Associated with Anxiety and Depression among Medical Students: An Online Survey**

**Supplementary Materials**

[**Table S1.** Spearman’s correlation (rho) between independent variables among medical students (n= 5765). 2](#_Toc79283221)

# **Table S1.** Spearman’s correlation (rho) between independent variables among medical students (n= 5765).

| Variables | Age | Gender | Academic year | Ability  to pay | S-COVID-19-S | UHC | BMI | Smoking | Drinking | Physical activity | Eating habits | HES | FCOV-19 | HL |
| --- | --- | --- | --- | --- | --- | --- | --- | --- | --- | --- | --- | --- | --- | --- |
| Gender | -.054 |  |  |  |  |  |  |  |  |  |  |  |  |  |
| Academic year | **.815** | -.053 |  |  |  |  |  |  |  |  |  |  |  |  |
| Ability to pay | -.006 | .000 | .002 |  |  |  |  |  |  |  |  |  |  |  |
| S-COVID-19-S | -.053 | -.019 | -.059 | -.027 |  |  |  |  |  |  |  |  |  |  |
| UHC | .008 | .029 | -.001 | .008 | .094 |  |  |  |  |  |  |  |  |  |
| BMI | .022 | .168 | .007 | .020 | .011 | -.007 |  |  |  |  |  |  |  |  |
| Smoking | .033 | .104 | .031 | -.010 | .005 | .028 | .012 |  |  |  |  |  |  |  |
| Drinking | .059 | .085 | .064 | -.016 | .009 | .033 | .006 | .**490** |  |  |  |  |  |  |
| Physical activity | -.022 | -.030 | -.018 | .045 | -.026 | .002 | -.034 | .046 | .048 |  |  |  |  |  |
| Eating habits | -.020 | -.101 | -.040 | .028 | -.014 | -.030 | -.005 | -.034 | -.077 | .162 |  |  |  |  |
| HES | -.044 | .049 | -.037 | .059 | -.019 | .003 | -.019 | -.017 | -.050 | .031 | .074 |  |  |  |
| FCOV-19 | -.078 | -.072 | -.073 | -.060 | .017 | -.002 | .011 | .067 | .020 | -.002 | .040 | -.015 |  |  |
| HL | .170 | .090 | .165 | .143 | -.087 | -.007 | .030 | .056 | .019 | .057 | .065 | .153 | -.114 |  |
| DDL | .016 | .103 | .017 | .102 | -.083 | -.010 | .018 | .063 | .019 | .049 | .068 | .163 | -.047 | **.629** |

Abbreviations: S-COVID-19-S, COVID-19-like symptoms; UHC, underlying health conditions; HES, healthy eating score; HL, health literacy; DDL, digital healthy diet literacy.
